# A thank you to all our reviewers

**DOI:** 10.1308/rcsann.2024.0116

**Published:** 2025-01-01

We would like to take this opportunity to thank all our reviewers for the time and expertise you generously contribute to peer-reviewing articles submitted to the *Annals of the Royal College of Surgeons of England*. We want to acknowledge your support during 2024, without which it would not be possible to preserve the high standards of our journal.

Aber, Ahmed
Adil, Md Tanveer
Agrawal, A.
Ahmed, Ahmed
Akula, Bharathi
Albani, Agustin
Almidani, Ammar
Alnaib, Mustafa
Alsharkawy, Kareem
Ananth, Sankar
Asher, John
Asif, Aqua
Baker, Daryll
Barbur, Sabina
Barnard, James
Bater, Michael
Batten, Timothy
Bedford, James
Bellringer, Simon
Benson, John
Beswick, William
Blackburn, Julia
Bloch, Benjamin
Bowrey, David
Britton, David
Buchanan, Malcolm A
Buchanan, Gordon
Burke, Joshua
Burnand, Katherine
Carey, John
Chan, David
Chan, Gareth
Chapman, Mark
Claireaux, Henry
Coleman, Mark
Courtney, Michael
Cripps, Neil
Davies, Evan
De Jong, Mechteld
de Joseph, Jomon
Dearing, J
Desai, Mital
Di Marco, Aimee
Donne, Adam
Dwerryhouse, Simon
Earnshaw, Peter
El-Sharkawy, Ahmed
Emara, Khaled
Estefan, Martin
Evans, Jonathan
Fernando, Bimbi
Fleming, Myles
Fleming, Simon
Forshaw, Matthew
Ganau, Mario
Gómez- Ramírez, Joaquin
Goodacre, Tim
Griffiths, Ewen
Hance, Julian
Hancorn, Kate
Harikrishnan, Athur
Harvey, James
Hassan, Iram
Hawkins, Will
Holbrook, Charlotte
Hubbard, Thomas
Islam, Shofiq
Issa, Eyad
James, Leroy
James, Nick
Jarral, Reza
Jiao, Long
Jiwani, Mateen
Kershaw, Steven
Khan, Omar
Khong, Grace
Knowles, Charles
Kubba, Haytham
Lau, Andrew
Lockwood, Sonia
Loftus, Ian
Lotto, Attilio
Lovegrove, Richard
Lyons, Marie
Mactier, Mhairi
Manjaly, Joseph
Markey, Anne
Markham, Hannah
Maxwell-Armstrong, Charles
McAleer, Joseph
McBrearty, Anthony
McIrvine, Andrew
Milinis, Kristijonas
Miller, Andrew
Mishra, Ami
Mishra, Ankit
Misra, Nikhil
Morgan, Rheinallt
Murphy, Richard
Mutimer, Jonathan
Nabulyato, William
Nadarajah, Vicki
Nagala, Sidhartha
Nandra, Rajpal
O'Leary, Fionnula
Packham, Andrew
Paranathala, Menaka
Phillips, Jonathan
Pietropaolo, Amelia
Pitt, James
Quinlan, John
Ramsay, George
Ravichandran, Duraisamy
Rees, Rowland
Riffat, Faruque
Robertson, Andrew
Roy, Holly
Salem, Joe
Samant, Ambaresh
Sargazi, Nastaran
Savva, Constantinos
Senapati, A.
Sharma, Sunil
Sharpe, Ian
Siddiqui, Zohaib
Simpson, Gregory
Singh, Arvind
Sinha, Maneesh
Skaife, Paul
Skillman, Joanna
Slade, Dominic
Smith, C. D.
Smith, Frank
Smith, Henry
Sodergren, Mikael
Sohail, Irfan
Somaiya, Pranav
Standring, Susan
Stevenson, Andrew
Tillotson, Laura
Tozer, Phil
Uppal, Harpal
Walijee, Hussein
Wallace, Angus
Walsh, Ciaran
Wheeler, Robert
Whittingham-Jones, Paul
Wijesinghe, Lasantha
Williams, Andrew
Williams, Gethin
Williams, Mark
Williams, Nigel
Williams, Stephen
Wyld, Linda
Yardley, Iain
Zaver, Vasudev

